# Mitochondrial calcium overload is the trigger for carbon monoxide neurotoxicity

**DOI:** 10.1038/s41419-025-08012-1

**Published:** 2025-10-21

**Authors:** Plamena R. Angelova, Artyom Y. Baev, Sergey O. Bachurin, Isabella Myers, Andrey Y. Abramov

**Affiliations:** 1 Department of Clinical and Movement Neurosciences, UCL Queen Square Institute of Neurology, London, UK; 2 Laboratory of Experimental Biophysics, Centre for Advanced Technologies, Tashkent, Uzbekistan; 3 Department of Biophysics, Faculty of Biology, National University of Uzbekistan, Tashkent, Uzbekistan; 4 Institute of Physiologically Active Compounds, Russian Academy of Sciences, Chernogolovka, Moscow region Russia

**Keywords:** Mechanisms of disease, Cell death in the nervous system

## Abstract

Carbon monoxide is an important gasotransmitter and regulator of cell function in different tissues, including the central nervous system. However, in large doses, it is a poisonous gas that causes mortality and morbidity. Moreover, the majority of survivors of high-dose exposures develop serious neurological conditions. Here, we studied the effect of toxic concentrations of carbon monoxide released from the compound CORM-401 and its removal (re-oxygenation) on calcium signalling in primary cortical neurons and astrocytes. We found that CO induces changes in intracellular Ca^2+^ concentration in both neurons and astrocytes. The mechanism of these signals was different—in neurons, it was activated by NMDA and AMPA receptors, while in astrocytes, CO-induced fusion of VNUT2-positive vesicles followed by activation of P2Y receptors. Calcium signal in neurons and astrocytes promotes mitochondrial calcium uptake, which dramatically increases after the removal of CO from the medium, which, in combination with higher rates of production of ROS, induces mitochondrial permeability transition and cell death. CO-induced death of neurons and astrocytes could be prevented with partial inhibition of mitochondrial calcium uptake by Tg2112x and/or inhibition of ROS production in the phase of re-oxygenation. Thus, the bidirectional interaction between mitochondrial calcium overload and production of reactive oxygen species is crucial for CO-induced death of neurons and astrocytes.

## Introduction

Carbon monoxide (CO) is a colorless, odorless, tasteless gas which, at high levels, is poisonous. It is formed during the incomplete combustion of fossil fuels and wood. Despite improvements in domestic appliance safety and sensor technology in the form of CO alarms, accidental carbon monoxide toxicity is still considered an important domestic and workplace poison, causing mortality and morbidity. One of the significant outcomes of CO poisoning is that the majority of survivors (up to 70%) develop delayed neurological sequelae, including cognitive decline and movement disorders [1].

Carbon monoxide is also produced endogenously in mammalian cells in heme oxygenase during the essential reaction of heme catabolism [2, 3]. For a long time, CO was considered to be a signaling molecule in a number of tissues, including those of the central nervous system. The biological function of CO is mostly related to other gaseous molecules, where it plays a crucial role in vascular homeostasis and regeneration by improving endothelial function [4]. At physiological concentrations, CO regulates energy metabolism [5, 6]. As a signaling molecule, CO modifies the activity of calcium channels [7–9] and induces a calcium signal in epithelial cells [10].

Carbon monoxide toxicity is, in part, induced by the deprivation of oxygen to the tissues due to the formation of deoxyhaemoglobin and the induction of chemical anoxia by the inhibition of mitochondrial cytochrome c [11]. Additionally, one of the major disruptive effects of high levels of CO becomes evident at the time of oxygen reintroduction [12]. In brain cells—neurons and astrocytes—toxic concentrations of CO induce overproduction of reactive oxygen species (ROS) from different sources, with maximal oxidative effect seen at the time of re-oxygenation [12, 13]. Activation of the ROS-producing enzyme NADPH oxidase is triggered by a CO-induced calcium signal in brain cells. However, the mechanism of these changes in intracellular calcium concentration ([Ca^2+^]_c_) and whether this process is involved in the mechanism of CO-induced cell death is not yet clear.

Ca^2+^ controls almost all major processes in the cells, from development and division to cell death. In excitable cells such as neurons (and in part astrocytes), Ca^2+^ plays an important role in signal transduction. Mitochondria are key players in intracellular calcium signaling, which acts as a short-term Ca^2+^ buffer [14]. Mitochondrial Ca^2+^ uptake is important for the activation of energy production, but overloading the mitochondria with Ca^2+^ in combination with an increase in production of ROS induces the opening of the mitochondrial permeability transition pore (mPTP), which stimulates the release of pro-apoptotic proteins and activates cell death [15].

Here we studied the effect of toxic concentrations of CO (released from CORM-401) on the [Ca^2+^]_c_ of primary neurons and astrocytes. We found that CO triggered a high calcium signal in brain cells: in neurons, this was caused by the activation of glutamate receptors, while in astrocytes, it was caused by the release of ATP by the activation of P2Y receptors by VGLUT vesicle release. In mitochondria, a CO-induced calcium signal elevates Ca^2+^, with a massive calcium overload seen as CO is removed from the medium. A combination of higher rates of ROS production and mitochondrial calcium overload activates the opening of the mPTP and leads to cell death. The importance of mitochondrial Ca^2+^ increase in the mechanism of CO-induced cell death was also confirmed by pre-treatment of neurons and astrocytes with the partial inhibitor of mitochondrial calcium uptake Tg2112x, which protected neurons and astrocytes from CO-induced apoptosis and necrosis.

## Materials and methods

### Cell culture preparation

Mixed cortical neuroglial cultures were prepared from newborn rat pups (1–3 days) as described earlier [16]. Animal husbandry and experimental procedures were performed in full compliance with the United Kingdom Animal (Scientific Procedures) Act of 1986. Subjects were culled using Schedule 1 procedures, decapitation and whole brains were removed and placed in a Petri dish filled with ice-cold Ca^2+^, Mg^2+^-free HBSS solution under sterile conditions. Meninges were gently removed, cortex was dissociated from other brain parts and cut into small (1 mm) pieces with scissors and placed in 0.25% Trypsin solution for 15 min in a water bath at 37 °C, with gentle stirring once per minute. After trypsinization, the material was washed twice with Neurobasal A medium and dissociated using a fire-polished pipette. The suspension was left for 1 min to allow large pieces to settle on the bottom of the microtube. The cell suspension was transferred to another microtube and centrifuged for 2 min at 500×*g*. Supernatant was removed, and the pellet resuspended in culture medium. Cells were placed on round (25 mm diameter) coverslips covered with poly-D-lysine to allow cells to attach. The medium was then replaced with full Neurobasal A medium (supplemented with B-27, 2 mM GlutaMAX and Penicillin–Streptomycin). Cells were maintained at 37 °C in a humidified atmosphere of 5% CO_2_. Twice a week, half of the culture medium was refreshed. Cells were used for experiments between 14 and 18 days.

### Transfection and transduction

We next identified putative ATP-containing vesicles by transducing astrocytes to express eGFP-tagged vesicular nucleotide transporter (VNUT) (eGFP–VNUT) [17, 18].

For identification of the vesicular release, primary cultures transduced to express VNUT–eGFP were used to detect vesicular fusion events in astrocytes expressing VNUT–eGFP, as described in detail previously [18]. Viral vector was added to the incubation medium after 1 week of culturing (AVV–eGFAP–VNUT–GFP). Fluorescence was excited at 488 nm and collected at 500–530 nm for eGFP, and fusion events were recorded using ZEISS LSM 980 at 63x objective and using ZEISS Blue software.

Similarly, mitoGCaMP or ER-GCaMP constructs were transfected into primary cultures using Effectene and fluorescence was excited at 488 nm and collected at 500–530 nm using ZEISS LSM 980 at ×40 objective and data acquisition and analysis were done using ZEISS Blue software.

### Imaging of intracellular calcium concentration and mitochondrial membrane potential

For intracellular calcium concentration measurement, cells were loaded with 5 µM fura-2 AM or fluo-4 AM, and with 10 µM of Rhodamine 123 for mitochondrial membrane potential, and 0.005% Pluronic in a HEPES-buffered salt solution composed of (in mM) 156 NaCl, 3 KCl, 2 MgSO_4_, 1.25 KH_2_PO_4_, 2 CaCl_2_, 10 glucose and 10 HEPES. pH was adjusted to 7.35 with NaOH. Cells were loaded in the dark at room temperature for 40 min with fura-2 AM and fluo-4 AM, and 15 min with rhodamine 123, and washed with HBSS prior to the experiment. Fluorescent Ca^2+^ and Δ*ψ*_m_ measurements using fura-2 and rhodamine 123 were obtained on an epifluorescence inverted microscope equipped with a ×20 fluorite objective. Changes in [Ca^2+^]_c_ were monitored in single cells using excitation light provided by a Xenon arc lamp, the beam passing through a monochromator at 340, 380 nm (Cairn Research, Kent, UK). Changes in Δ*ψ*_m_ were monitored using a 10 nm band pass filter centered at 490 nm for excitation. Emitted fluorescence light for both dyes was reflected through a 515 nm long-pass filter to a cooled CCD camera (Retiga, QImaging, Canada) and digitized to 12-bit resolution.

For Fluo-4 based measurement of [Ca^2+^]_c_ the cells were excited by illumination at 488 nm, the fluorescence light was collected above 505 nm either by an epifluorescence inverted microscope equipped with a ×20 fluorite objective (Cairn Research, Kent, UK), a fluorescence-inverted microscope EVOS FL COLOR equipped with a ×20 objective, or Zeiss LSM 980 using ×40 oil immersion objective. Images for all experiments were collected at intervals of 10 s, digitized, and stored for off-line analysis using software from Andor (Belfast, UK).

### Imaging of mitochondrial and ER calcium

Confocal images were obtained using a Zeiss LSM 980 confocal laser scanning microscope using a ×40 oil immersion objective. The 488-nm Argon laser line was used to excite mitoGCaMP6f and ER-GCaMP6f fluorescence, which was measured at 505–550 nm. For Rhod-5N measurements, the 563 nm excitation and 580–630 nm emission were used.

All data presented were obtained from at least five coverslips and 2–3 different cell culture preparations.

### Cell death assay

To detect caspase-3/7 activation, cells were incubated with 5 μM NucView^TM^ 488 and 5 μM NucView^TM^ 488 Caspase-3 Substrate (NucView 488, Biotium, CA, USA) (excitation laser 488 nm with emission above 515 nm). Images were obtained using a Zeiss LSM 980 confocal microscope using a ×40 oil immersion objective as described in ref. [19]. The activation of mPTP opening and apoptosis in all cells was induced by the calcium ionophore Ferutinin (100 μM) [20, 21] and the number of NucView488 positive cells after Ferutinin application was taken as 100%.

The level of cell death was evaluated with the help of propidium iodide and Hoechst 33342. Hoechst stains chromatin DNA of all available cells. As propidium iodide is only permeable to dead cells, it is possible to calculate the percentage of dead cells (showing red fluorescence of propidium iodide) versus the total number of cells (showing blue fluorescence of Hoechst).

Cells were loaded for 15 min with 20 μM propidium iodide and 10 μM Hoechst 33342. Fluorescence measurements were obtained on an epifluorescence-inverted microscope equipped with a ×20 fluorite objective. Excitation light for Hoechst 33342 (380 nm) and for PI (530 nm) was provided by a Xenon arc lamp. Emitted fluorescence light was reflected through a 515-nm long-pass filter to a cooled CCD camera (Retiga, QImaging, Canada).

### Statistical analysis

Statistical analysis and curve data fitting were performed using OriginPro 2022 (Microcal Software Inc., Northampton, MA, USA). Experimental data are shown as mean ± SEM. Two-sample *t*-test was used to analyze the differences between two groups, and a one-way ANOVA with post hoc Tukey test was used to analyze the differences between three or more groups. Differences between groups were considered significant if *p* ≤ 0.05.

## Results

### CO induces calcium signal in neurons and astrocytes

**Fig. 1:**
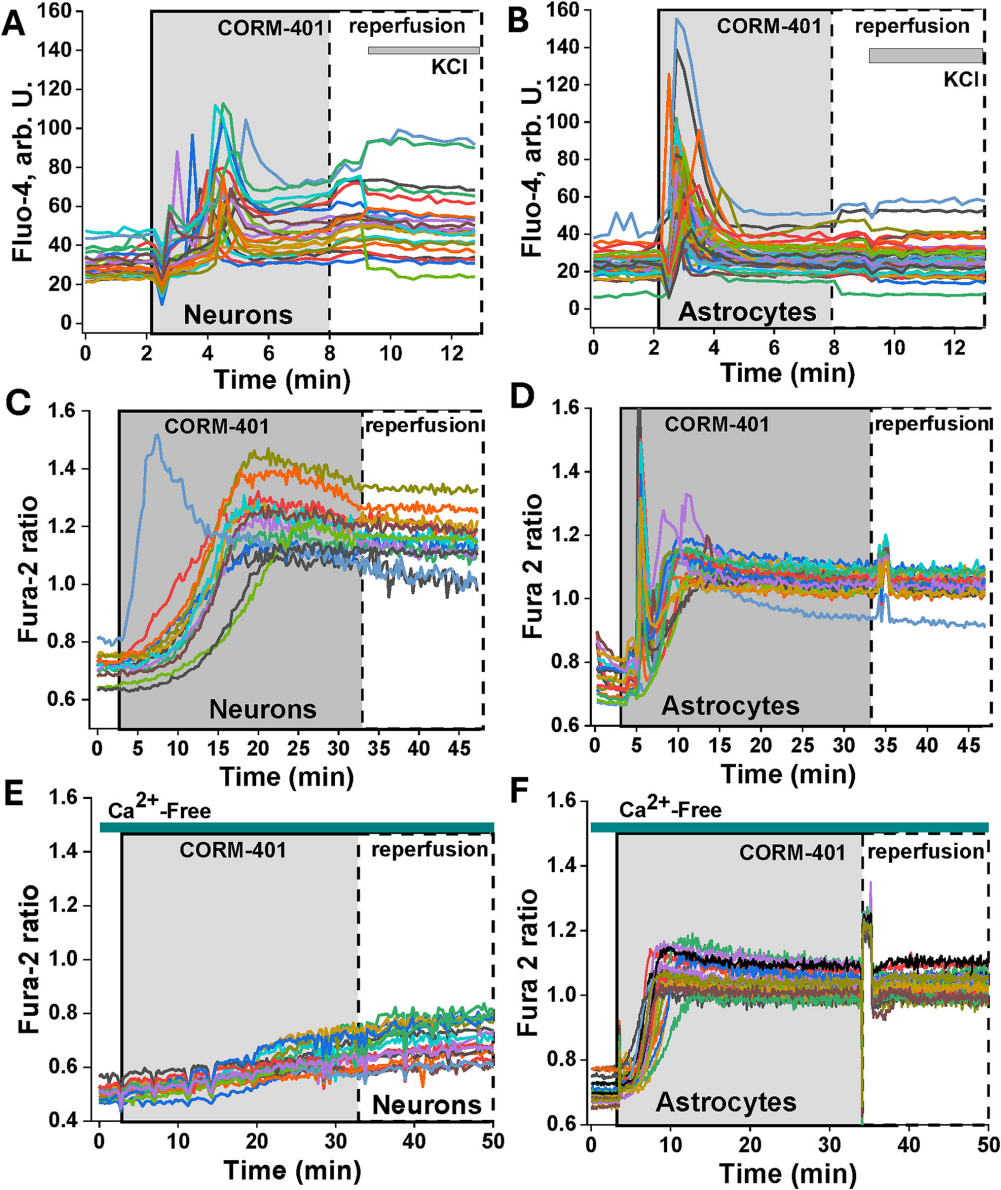
CO induces [Ca^2+^]_c_ increase in primary cortical neurons and astrocytes. 60 μM CORM-401 induces calcium signal in neurons (**A**, **C**) and astrocytes (**B**, **D**) measured by fluorescent indicators fluo-4 (**A**, **B**) and fura-2 ratio (340/380 nm; **C**–**F**). Ca^2+^-free medium (plus 0.5 mM EGTA) blocks calcium signal in neurons (**E**) but not in astrocytes (**F**). 50 mM KCl was added to induce a calcium signal in neurons. The single traces correspond to measurements of fluorescence from single cells.

Application of the CORM-401 (60 μM) to primary cortical co-culture neurons and astrocytes loaded with fluorescent indicators for Ca^2+^-Fluo-4 or Fura-2, induced a profound increase of intracellular calcium concentration ([Ca^2+^]_c_) in both neurons and astrocytes (*n* = 215 neurons; *n* = 309 astrocytes; Fig. 1A, B). Elevated [Ca^2+^]_c_ was observed in the majority of cells during CO exposure. In a small proportion of cells, it was increased with re-oxygenation (15 ± 1% of neurons, 10 ± 1% astrocytes; Fig. 1A, B). The amplitude and the shape of the CO-induced calcium signal in neurons and astrocytes varied from a fast and transient signal to a slow increase in [Ca^2+^]_c_ (Fig. 1C, D). In the majority of experiments, neurons were also identified functionally following the application of 50 mM KCl. This induced depolarization and stimulation of calcium flux through voltage-dependent Ca^2+^ channels and voltage-gated receptors in neurons (Fig. 1A–C).

CO-induced calcium signal in neurons and astrocytes has a different source of Ca^2+^. Thus, Ca^2+^-free medium (plus 0.5 mM EGTA) significantly reduced the effect of CO on the [Ca^2+^]_c_ of neurons (*n* = 96; Fig. 1E), while in astrocytes, the calcium signal was almost unchanged (*n* = 145; Fig. 1F).

### CO-induced release of Ca^2+^ from endoplasmic reticulum in astrocytes

**Fig. 2:**
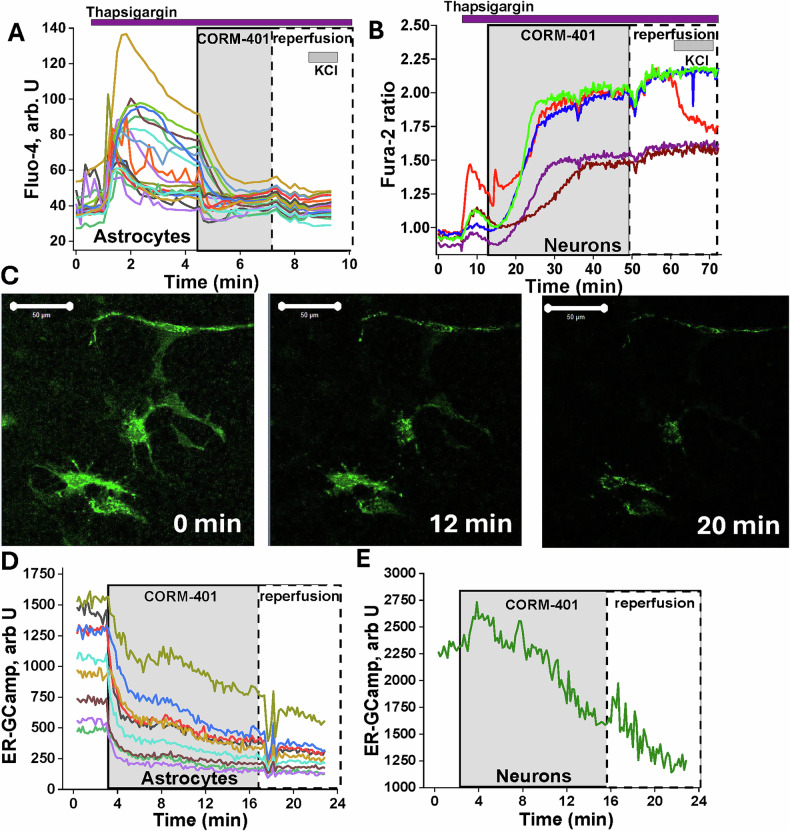
Carbon monoxide in astrocytes induces the release of Ca^2+^ from the endoplasmic reticulum. Inhibition of SERCA with 0.5 μM thapsigargin blocks CO-induced calcium signal in astrocytes (**A**) but not neurons (**B**). Measurements of Ca^2+^ concentration in ER with genetically-encoded indicator ER-GCaMP (**C**) showed that CO triggers the release of calcium from the ER in astrocytes (**D**) and, after a delay, partially in neurons (**E**). Scale bar is 50 μm. 50 mM KCl was added to induce a calcium signal in neurons. The single traces correspond to measurements of fluorescence from single cells.

The inhibitor of SERCA Thapsigargin (1 μM) induces the release of Ca^2+^ from the endoplasmic reticulum to cytosol, resulting in an increase in the [Ca^2+^]_c_ of neurons and astrocytes (*n* = 94 neurons; *n* = 111 astrocytes; Fig. 2A, B). Emptying of the calcium pool of ER with Thapsigargin completely blocked the CO-induced calcium signal in astrocytes and did not change [Ca^2+^]_c_ in these cells after re-perfusion (Fig. 2A). In contrast to astrocytes, incubation of neurons with 1 μM Thapsigargin did not block the CO-induced calcium signal in these cells (Fig. 2B).

To confirm ER-dependence of the CO-induced calcium signal in astrocytes but not in neurons, we used genetically encoded ER-GCaMP indicator in primary cortical neurons and astrocytes (Fig. 2C). Application of 60 μM CORM-401 to a co-culture of neurons and astrocytes with transfected ER-GCaMP induced a decrease in ER calcium in astrocytes (*n* = 56; Fig. 2D). In neurons, the effect of CORM-401 on ER-GCaMP fluorescence was much lower, although CO also induced a decrease in ER calcium in some neurons after a ~5–10 min delay (*n* = 33; Fig. 2E).

Thus, CO-induced calcium signal in astrocytes originates from the release of Ca^2+^ from ER, while in neurons this signal was dependent on entry of external Ca^2+^ to the cytosol.

### CO induces calcium signal in astrocytes through activation of P2Y receptors and phospholipase C

**Fig. 3:**
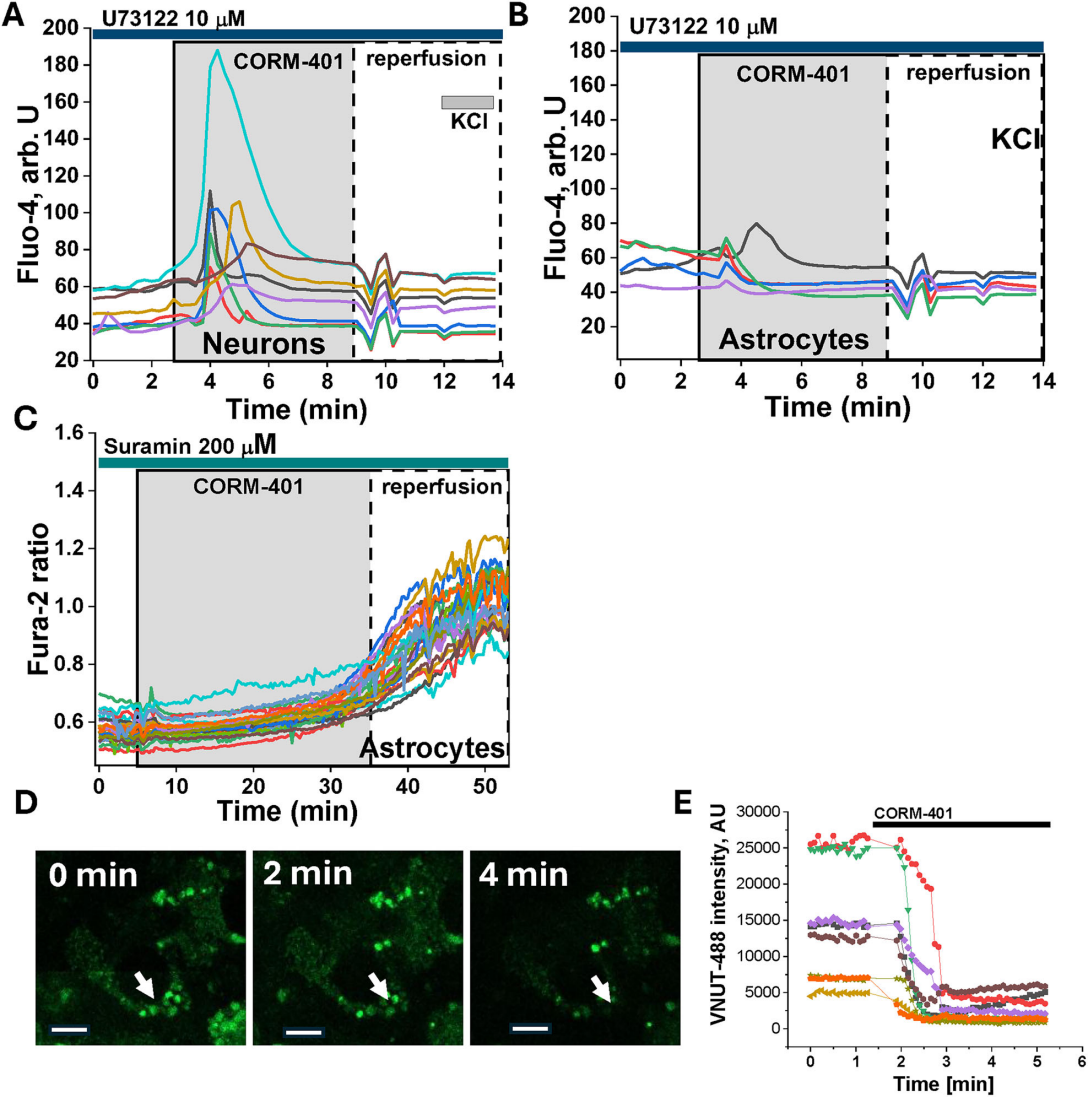
CO induces a calcium signal in astrocytes by activating P2Y receptors and phospholipase C. 20 min pre-incubation of primary co-culture of neurons and astrocytes with 10 μM U73122 inhibited CO-induced (CORM-401, 60 μM) calcium signal in astrocytes (**B**) but not in neurons (**A**). **C** Inhibitor of P2Y receptors Suramin (200 μM) blocked the initial Ca^2+^ signal during CO exposure. VNUT-GFP expression in cortical astrocytes (**D**) is fused with the time of CO exposure (**E**).

In astrocytes, the release of Ca^2+^ from the ER to the cytosol could be activated by IP3 receptors. IP3 is produced by the phospholipase C (PLC). To establish whether PLC is involved in the CO-induced calcium signal, we used cells pre-incubated (20 min) with an inhibitor of PLC-U73122 (10 μM). CO-induced elevation of [Ca^2+^]_c_ in the cortical astrocytes was almost completely inhibited (*n* = 82 cells; Fig. 3A). Considering that P2Y receptors can activate the PLC-IP3-related pathway, we then used 200 μM Suramin as a potent P2Y receptor blocker. We found that 20 min pre-incubation of the cells with this compound completely blocked the initial CO-induced calcium signal in astrocytes (*n* = 156 astrocytes; Fig. 3B) but not in neurons (*n* = 68; Fig. 3C). This strongly suggested carbon monoxide activates the P2Y receptor in astrocytes. It should be noted that astrocytes exposed to CO for a longer period (35–40 min) induced a slow and progressive [Ca^2+^]_c_ increase that coincided with ATP deprivation observed previously [13].

### CO induces fusion of the VNUT1 vesicles in astrocytes

Vesicular nucleotide transporter (VNUT)-expressing vesicles contain ATP and inorganic polyphosphate that could activate P2Y receptors [18]. 60 μM CORM-401 was applied to a primary co-culture of neurons and astrocytes with expression of GFP in astrocytic VNUT (Fig. 3D). Release of CO to the medium induced fast fusion of the VNUT-GFP vesicles in astrocytes (*n* = 83; Fig. 3D), strongly suggesting that a release of nucleotides or inorganic polyphosphate can be the trigger of a calcium signal via P2Y receptor activation.

### CO induces activation of glutamate receptors

**Fig. 4:**
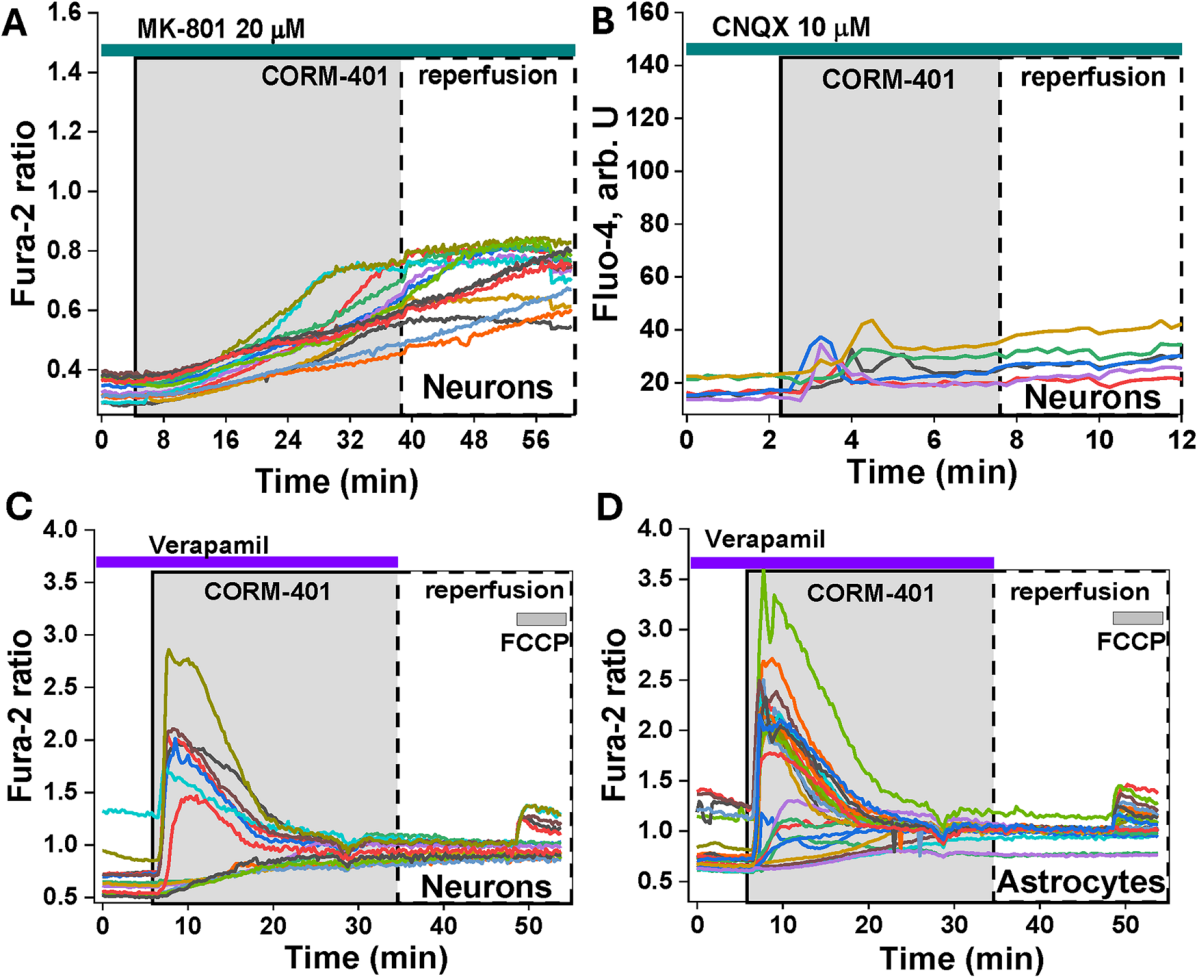
CO-induced calcium signal in primary cortical neurons is dependent on glutamate antagonists. Incubation of co-culture of neurons and astrocytes with inhibitors of NMDA receptor (**A**, 20 μM MK-801) or AMPA receptor (**B**, 10 μM CNQX) decreased the CO-induced calcium signal in neurons. Inhibitor of VDCC 20 μM Verapamil had no effect on CO-induced calcium signal in neurons (**C**) and astrocytes (**D**).

In neurons, a CO-induced calcium signal is dependent on external Ca^2+,^ and in considering this, experiments focused on the major Ca^2+^ ion channels. Incubation of the primary co-culture of neurons and astrocytes with an inhibitor of NMDA receptors—20 μM MK-801 for 20 min, inhibited a CO-induced calcium signal (*n* = 114 neurons; Fig. 4A). Incubation of the cells with an inhibitor of another type of glutamate receptor—AMPA, 10 μM CNQX, also inhibited most of the CO-induced [Ca^2+^]_c_ elevation in neurons (*n* = 87; Fig. 4B). However, inhibition of voltage-dependent calcium channels (VDCC) with 20 μM verapamil did not change the amplitude or the shape of the CO-induced calcium signal in primary cortical neurons (Fig. 4C) and inhibitors of NMDA, AMPA and VDCC did not change CO-induced changes in [Ca^2+^]_c_ in astrocytes (Fig. 4D).

### CO-induced calcium signal increases [Ca^2+^]_m_ at the time of re-perfusion and activates caspase-3

**Fig. 5:**
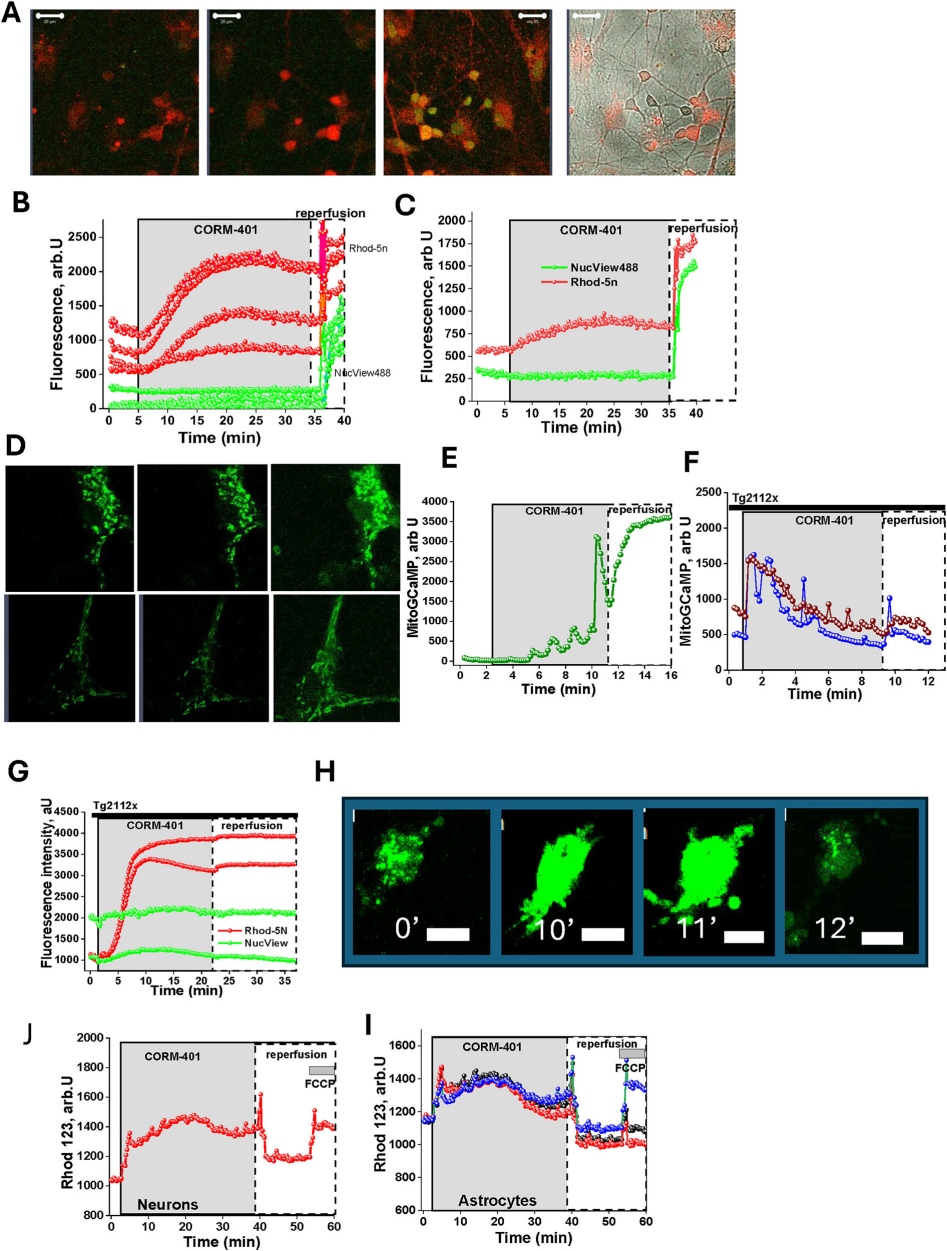
Carbon monoxide induces mitochondrial calcium overload and caspase-3 activation. **A** Rhod-5n and Nuc-view488 images before and after application of 60 μM CORM-401. Bars are 20 μm. **B**, **C** Effect of CO on mitochondrial Ca^2+^ and caspase 3, red lines -Rhod-5n, green line—NucView488 fluorescence. Measurements of [Ca^2+^]_m_ in cortical neurons and astrocytes with mitoGCaMP (**D**) during 60 μM CORM-401 application and reperfusion (**E**) in the presence of Tg-2112x (1 μM) (**G**). **F** Tg2112x does not block cytosolic calcium signal but reduces mitochondrial calcium uptake and inhibits CO-induced caspase-3 activation. **H** Effect of 1 μM Cyclosporin A on CO-induced calcium changes in neurons (mitoGCaMP). 60 μM CORM-401 induces profound mitochondrial depolarization (Rhodamine123 fluorescence) in primary cortical neurons (**J**) and astrocytes (**I**), which is recovered after reperfusion. Mitochondrial uncoupler 1 μM FCCP was added at the end of the experiment to induce complete mitochondrial depolarization.

Mitochondria play the role of a short-term calcium buffer in brain cells, and calcium overload of mitochondria in combination with reactive oxygen species (ROS) production may trigger mitochondrial permeability transition, followed by cell death [22]. Application of CORM-401 (60 μM) to primary cortical neurons and astrocytes loaded with the low affinity calcium indicator Rhod-5N (which due to its charge is loaded mainly in mitochondria) induces a slow and progressive increase of Rhod-5N fluorescence in both cytosol and mitochondrial compartments, with a higher calcium increase seen in the cytosol of neurons and astrocytes (Fig. 5A, B; *N* = 6 experiments; *n* = 125 neurons; *n* = 88 astrocytes). Interestingly, washing CORM-401 out of the medium induced a fast and profound increase of Ca^2+^ signal in mitochondria (Fig. 5A, B). It should be noted that Rhod-5N is a low-affinity indicator, suggesting there is already a pathological calcium overload in these organelles. Simultaneous measurement of Rhod-5N with the fluorescent substrate for caspase-3—NucView^TM^ 488 in these cells showed a rapid increase of the signal at the time of rapid mitochondrial calcium increase that coincided with reperfusion (Fig. 5A–C). This strongly suggests activation of the process of apoptosis in these cells.

Using primary neurons and astrocytes with the genetically encoded mitochondrial Ca^2+^-mitoGCaMP, we found that application of CORM-401 (60 μM) induced relatively low changes in [Ca^2+^]_m_ compared to cytosolic signal (Fig. 5D, E), which is dramatically increased after removing CO from the medium (Fig. 5D, E). Previously, we discovered a compound that limits but does not completely inhibit mitochondrial calcium uptake, thus successfully protecting neurons against calcium-dependent cell death in glutamate excitotoxicity and in β-amyloid-induced neuronal loss [23, 24]. Pre-incubation of the neurons and astrocytes with 10 μM Tg2112x did not induce changes in [Ca^2+^]_m_ of astrocytes or neurons after application of CORM-401 (60 μM) but significantly reduced mitochondrial Ca^2+^ uptake following removal of CO from the medium (Fig. 5F). Pre-treatment of the neurons and astrocytes loaded with Rhod-5N and NucView^TM^ 488 with Tg2112x (20 min, 10 μM) completely blocked the activation of caspase-3 (shown by NucView^TM^488 fluorescence rise) in these cells after application and subsequent removal of the 60 μM CORM-401 (Fig. 5G). However, in considering the mitochondrial and cytosolic distribution of the Rhod-5N, changes in fluorescence of this indicator in mitochondria at the time of washing CORM-401 off from the medium were not observed (Fig. 5G), although it was confirmed that Tg2112x had no effect on CO-induced cytosolic elevation of Ca^2+^. In considering the mitochondrial calcium overload at the time of re-oxygenation and activation of caspase-3, it is suggested that neuronal death could be induced by the opening of the mitochondrial permeability transition pore in these cells. Pre-treatment of the neurons and astrocytes with an inhibitor of mPTP—1 μM Cyclosporine A (CsA), did not change the CO-induced calcium signal in mitochondria or cytosol, but did block the increase of NucView^TM^488 fluorescence in these cells (Fig. 5H), confirming that caspase-3 activation and apoptosis are induced by the opening of the mPTP. To explain the massive calcium uptake and mPTP opening at the time CORM-401 is removed from the medium and while [Ca^2+^]_c_ is elevated, mitochondrial membrane potential was measured at the time of CO application using Rh123 as a fluorescent indicator. In agreement with previously published data [13], we have found that the application of 60 μM CORM-401 induced profound mitochondrial depolarization (88 ± 9%, *n* = 111 neurons and 142 astrocytes; Fig. 5J, I), which could be restored at the time of re-oxygenation (Fig. 5H), suggesting a massive uptake of Ca^2+^ by mitochondria at the time of re-oxygenation when Δψm is partially restored.

### Sequestration of mitochondrial Ca^2+^ uptake inhibits CO-induced necrotic cell death in neurons and astrocytes

CO was able to induce not only apoptotic cell death but also necrosis in neurons and astrocytes [13]. Guided by previous experiments, where pre-incubation of the primary co-culture of neurons and astrocytes with various inhibitors modified the calcium signal, the role of the calcium signal in CO-induced cell death was studied.

**Fig. 6:**
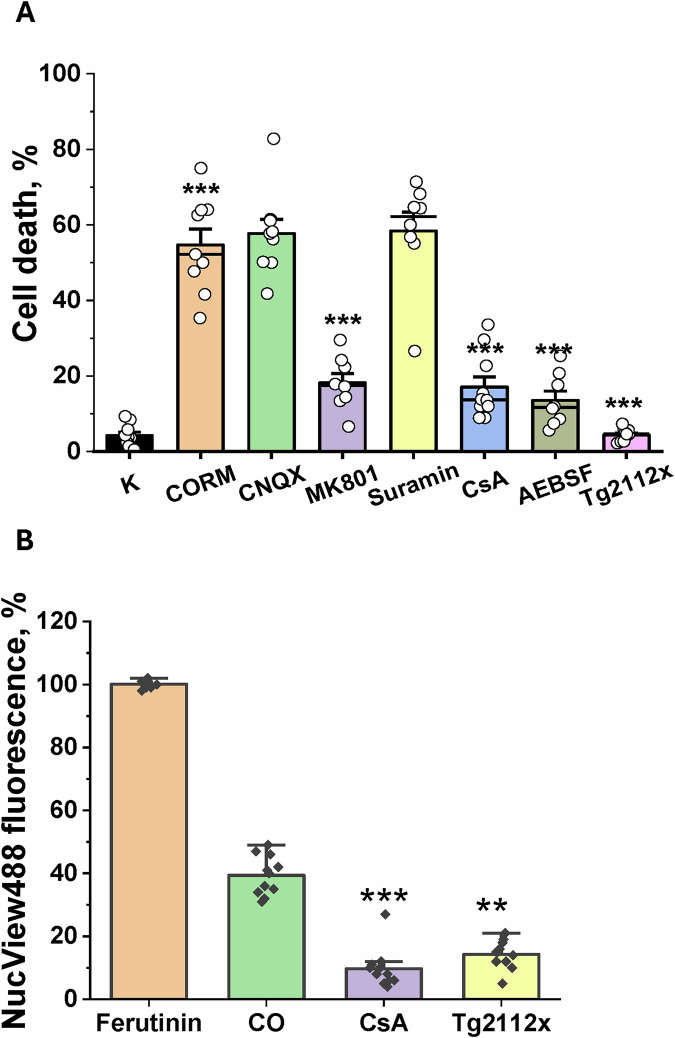
CO-induced neurotoxicity could be prevented by the inhibition of NMDA receptors and the inhibition of mitochondrial calcium uptake. **A** Cell death percentage was assessed in the primary co-culture of neurons and astrocytes upon the application of 60 μM CORM-401/reoxygenation and after pre-treatment with various enzymes and receptors. Cell death represents the percentage of propidium iodide (PI)-positive cells (dead cells) vs. Hoechst-positive cells (total cell number). **B** Percentage of NucView488-positive cells was assessed in primary co-culture of neurons and astrocytes upon application of 60 μM CORM-401/reoxygenation and after pre-treatment with 1 μM Cyclosporin C or 1 μM Tg2112x, normalized to the response to the effect of 100 μM Ferutinin. Data are represented as mean ± SEM. ∗∗∗*p* < 0.0001; **p < 0.001.

40 min incubation of the primary co-culture of cortical neurons and astrocytes with 60 μM CORM-401 followed by washing CO out of the medium and a 24-h period before measurement of the level of cell death, induced necrosis in 55% of the cells (Fig. 6A). In astrocytes, pre-incubation of the cells with inhibitors of CO-induced calcium signal–suramin, an inhibitor of the AMPA receptors CNQX, did not change the percentage of dead cells (Fig. 6A). However, the inhibitor of NMDA receptor-MK-801 significantly reduced the number of dead neurons and astrocytes (Fig. 6A) suggesting the importance of calcium signal in CO-induced toxicity. Importantly, partial inhibition of the mitochondrial Ca^2+^ uptake with 1 μM Tg2112x almost completely protected neurons and astrocytes against CO-induced cell death (Fig. 6A), pointing towards the importance of mitochondrial calcium overload in the mechanism of CO-induced toxicity. Mitochondrial mPTP inhibitor Cyclosporin A also inhibited cell death induced by CO, suggesting that mitochondrial calcium overload plus ROS overproduction triggers mPTP opening and induces cell death (Fig. 6A). Noting that not mitochondrial calcium overload but ROS overproduction can be blocked by inhibition of NADPH oxidase with 20 μM AEBSF.

In order to quantify the effect of CO treatment on caspase-3/apoptosis activation, we used the data from the figures presented in Fig. 5 and normalized it to the total caspase 3/apoptosis activation triggered by electrogenic calcium ionophore Ferutinin (100 μM) that overloads mitochondria and induces mPTP opening and apoptosis [20, 21]. We have found that 60 μM CORM-401 and 15 min reperfusion induce activation of apoptosis in 39 ± 6% of cells (Fig. 6B). This effect was associated with mitochondrial calcium overload and opening of mPTP because it could be reduced by pre-treatment of neurons and astrocytes with 1 μM Cyclosporine A (CsA) or 1 μM Tg2112x (Fig. 6B).

## Discussion

Carbon monoxide toxicity is shown to be triggered by oxygen deprivation that leads to an energy collapse and oxidative stress [12, 13]. However, the central nervous system consists of excitable cells where processes of calcium signal transduction, redox balance and energy metabolism are closely related to each other [13, 25]. Here we show that toxic concentrations of CO induced an increase in [Ca^2+^]_c_ in both neurons and astrocytes. Importantly, the mechanisms of activation differ. Thus, in neurons, the calcium signal is dependent on the presence of external calcium in the medium, while astrocytes were more dependent on the Ca^2+^ of the intracellular store (Fig. 2). A CO-induced calcium signal in neurons was triggered by the activation of glutamate receptors—NMDA and AMPA. The initial trigger for this signal was the release of glutamate by CO or ischemia (Fig. 3). This confirms the importance of glutamate excitotoxicity in ischemia-induced brain injury [26]. This type of calcium signal in neurons has been found previously to be an activator of NADPH oxidase in ischemia or CO-induced oxidative injury [13, 27]. Interestingly, an inhibitor of NMDA receptors—MK-801, was shown to be protective against CO-induced hearing loss [28]. There is the potential for the activation of glutamate receptors with high concentrations of glutamate to be toxic for neurons due to the induction of energy deprivation and a massive calcium overload (delayed calcium deregulation) [29, 30]. However, in the case of CO exposure, it is clear that this cannot be the only trigger for neuronal loss because it also kills astrocytes.

In astrocytes, CO induces fusion of VNUT vesicles, which contain ATP, ADP and inorganic polyphosphate, which can activate the calcium signal in astrocytes via P2Y receptors [18, 31, 32]. In our experiments, the P2Y antagonist not only inhibited a CO-induced calcium signal in astrocytes but also reduced the number of dead cells, highlighting the importance of the [Ca^2+^]_c_ elevation in astrocytes in the mechanism of CO-induced toxicity. However, in contrast to glutamate in neurons, activation of P2Y receptors in astrocytes does not induce cell death unless in combination with other mechanisms; they were even shown to have a protective effect against glutamate and ischemia [33, 34]. The higher-than-normal calcium increases in astrocytes and in neurons, which we found with low-affinity calcium indicator Rhod-5N, cannot be the result of only P2Y activation but may be associated with energy deprivation after prolonged CO exposure and inhibition of Ca^2+^-ATPases, which has also been shown in previous studies [13, 35].

CO-induced elevation of the [Ca^2+^]_c_ in neurons and astrocytes leads to mitochondrial Ca^2+^ uptake; a relatively low uptake during CO exposure, but which massively increases at the time of CO removal. Mitochondrial Ca^2+^ uptake is dependent on Δψm [14, 36]. During CO exposure depolarized mitochondria cannot accept calcium, but at the time of removal of the CORM-401 from the medium (i.e. removal of CO), mitochondrial membrane potential recovers, triggering the transport of high levels of Ca^2+^ to the mitochondria (Fig. 5). Mitochondrial Ca^2+^ uptake at the time of CO removal from the media is an important part in the mechanism of neuronal and astrocytic cell death. This is because both mitochondrial calcium transport and CO-induced cell death could be significantly reduced by the partial inhibitor of mitochondrial Ca^2+^ uptake, Tg2112x [23, 24].

Washing the CO out of the medium (replicating reoxygenation/ reperfusion of the cells) is the key trigger for the mechanism of cell death. Reoxygenation restores the function of the electron transport chain and Δ*ψ*_m_, which leads to a massive mitochondrial Ca^2+^ uptake that, at the same time, provides oxygen for the overproduction of superoxide and H_2_O_2_ in NADPH oxidases [13, 27]. The combination of high mitochondrial Ca^2+^ and a higher rate of reactive oxygen species production is the trigger for the opening of the mPTP, initiating the pathways of apoptosis and necrosis [15, 21, 37, 38]. The opening of mPTP and consequent brain cell death is the event we observed with CO reperfusion (Figs. 5, 6).

Thus, carbon monoxide toxicity in brain cells is triggered by a combination of glutamate- or ATP-induced calcium signaling, followed by mitochondrial calcium uptake at the time of reperfusion. This, in combination with ROS overproduction, leads to mPTP opening and cell death. Our data suggests that CO-induced neuronal damage has the potential to be significantly reduced by the introduction of inhibitors of mitochondrial calcium uptake and inhibitors of ROS producers before or at the time of re-introduction of oxygen. This finding has the potential to enhance current oxygen therapy practice following carbon monoxide exposure by preventing neuronal damage. What this reduction in neuronal damage might mean for the delayed neurological sequelae often seen following carbon monoxide poisoning, or for the pathogenesis of neurodegenerative disorders that could be associated with carbon monoxide poisoning, requires further investigation.

## Data Availability

All data supporting the conclusions of this manuscript are provided in the text and figures. Data available upon request.

## References

[1] Oh S, Choi SC. Acute carbon monoxide poisoning and delayed neurological sequelae: a potential neuroprotection bundle therapy. Neural RegenRes. 2015;10:36–8. 10.4103/1673-5374.150644 PMC4357109 25788913

[2] Tenhunen R, Marver HS, Schmid R. The enzymatic conversion of heme to bilirubin by microsomal heme oxygenase. Proc Natl Acad Sci USA. 1968;61:748–55. 4386763 10.1073/pnas.61.2.748 PMC225223

[3] Campbell NK, Fitzgerald HK, Dunne A. Regulation of inflammation by the antioxidant haem oxygenase 1. Nat Rev Immunol. 2021;21:411–25. 33514947 10.1038/s41577-020-00491-x

[4] Kim YM, Pae HO, Park JE, Lee YC, Woo JM, Kim NH, et al. Heme oxygenase in the regulation of vascular biology: from molecular mechanisms to therapeutic opportunities. Antioxidants Redox Signal. 2011;14:137–67. 10.1089/ars.2010.3153 PMC2988629 20624029

[5] Kaczara P, Motterlini R, Kus K, Zakrzewska A, Abramov AY, Chlopicki S. Carbon monoxide shifts energetic metabolism from glycolysis to oxidative phosphorylation in endothelial cells. FEBS Lett. 2016;590:3469–80. 27670394 10.1002/1873-3468.12434

[6] Almeida AS, Figueiredo-Pereira C, Vieira HL. Carbon monoxide and mitochondria-modulation of cell metabolism, redox response and cell death. Front Physiol. 2015;6:33. 25709582 10.3389/fphys.2015.00033 PMC4321562

[7] Boycott HE, Dallas ML, Elies J, Pettinger L, Boyle JP, Scragg JL, et al. Carbon monoxide inhibition of Cav3.2 T-type Ca^2+^ channels reveals tonic modulation by thioredoxin. FASEB J. 2013;27:3395–407. 23671274 10.1096/fj.13-227249

[8] Wilkinson WJ, Kemp PJ. Carbon monoxide: an emerging regulator of ion channels. J Physiol. 2011;589:3055–62. 21521759 10.1113/jphysiol.2011.206706 PMC3145923

[9] Peers C. Ion channels as target effectors for carbon monoxide. Exp Physiol. 2011;96:836–9. 21551266 10.1113/expphysiol.2011.059063

[10] Kaczara P, Proniewski B, Lovejoy C, Kus K, Motterlini R, Abramov AY, et al. CORM-401 induces calcium signalling, NO increase and activation of pentose phosphate pathway in endothelial cells. FEBS J. 2018;285:1346–58. 29464848 10.1111/febs.14411

[11] Miro O, Casademont J, Barrientos A, Urbano-Marquez A, Cardellach F. Mitochondrial cytochrome c oxidase inhibition during acute carbon monoxide poisoning. Pharmacol Toxicol. 1998;82:199–202. 9584335 10.1111/j.1600-0773.1998.tb01425.x

[12] Abramov AY, Myers I, Angelova PR. Carbon monoxide: a pleiotropic redox regulator of life and death. Antioxidants (Basel). 2024;13:1121. 10.3390/antiox13091121 PMC11428877 39334780

[13] Angelova PR, Myers I, Abramov AY. Carbon monoxide neurotoxicity is triggered by oxidative stress induced by ROS production from three distinct cellular sources. Redox Biol. 2023;60:102598. 36640724 10.1016/j.redox.2022.102598 PMC9852609

[14] Abeti R, Abramov AY. Mitochondrial Ca^2+^ in neurodegenerative disorders. Pharmacol Res. 2015;99:377–81. 26013908 10.1016/j.phrs.2015.05.007

[15] Baev AY, Vinokurov AY, Potapova EV, Dunaev AV, Angelova PR, Abramov AY. Mitochondrial permeability transition, cell death and neurodegeneration. Cells. 2024;13:1–20. 10.3390/cells13070648 PMC11011324 38607087

[16] Holmstrom KM, Marina N, Baev AY, Wood NW, Gourine AV, Abramov AY. Signalling properties of inorganic polyphosphate in the mammalian brain. Nat Commun. 2013;4:1362. 23322050 10.1038/ncomms2364 PMC3562455

[17] Sawada K, Echigo N, Juge N, Miyaji T, Otsuka M, Omote H, et al. Identification of a vesicular nucleotide transporter. Proc Natl Acad Sci USA. 2008;105:5683–6. 18375752 10.1073/pnas.0800141105 PMC2311367

[18] Angelova PR, Iversen KZ, Teschemacher AG, Kasparov S, Gourine AV, Abramov AY. Signal transduction in astrocytes: localization and release of inorganic polyphosphate. Glia. 2018;66:2126–36. 30260496 10.1002/glia.23466 PMC6282517

[19] Novikova IN, Potapova EV, Dremin VV, Dunaev AV, Abramov AY. Laser-induced singlet oxygen selectively triggers oscillatory mitochondrial permeability transition and apoptosis in melanoma cell lines. Life Sci. 2022;304:120720. 35716733 10.1016/j.lfs.2022.120720

[20] Abramov AY, Duchen MR. Actions of ionomycin, 4-BrA23187 and a novel electrogenic Ca^2+^ ionophore on mitochondria in intact cells. Cell calcium. 2003;33:101–12. 12531186 10.1016/s0143-4160(02)00203-8

[21] Kritskaya KA, Stelmashchuk OA, Abramov AY. Point of no return—what is the threshold of mitochondria with permeability transition in cells to trigger cell death. J Cell Physiol. 2025;240:e31521. 39760157 10.1002/jcp.31521 PMC11701880

[22] Baev AY, Vinokurov AY, Novikova IN, Dremin VV, Potapova EV, Abramov AY. Interaction of mitochondrial calcium and ROS in neurodegeneration. Cells. 2022;11:1–17. 10.3390/cells11040706 PMC8869783 35203354

[23] Angelova PR, Vinogradova D, Neganova ME, Serkova TP, Sokolov VV, Bachurin SO, et al. Pharmacological sequestration of mitochondrial calcium uptake protects neurons against glutamate excitotoxicity. Mol Neurobiol. 2019;56:2244–55. 30008072 10.1007/s12035-018-1204-8 PMC6394642

[24] Shevtsova EF, Angelova PR, Stelmashchuk OA, Esteras N, Vasil’eva NA, Maltsev AV, et al. Pharmacological sequestration of mitochondrial calcium uptake protects against dementia and beta-amyloid neurotoxicity. Sci Rep. 2022;12:12766. 35896565 10.1038/s41598-022-16817-9 PMC9329451

[25] Angelova PR, Abramov AY. Interplay of mitochondrial calcium signalling and reactive oxygen species production in the brain. Biochem Soc Trans. 2024;52:1939–46. 39171662 10.1042/BST20240261 PMC11668289

[26] Rothman SM, Olney JW. Glutamate and the pathophysiology of hypoxic–ischemic brain damage. Ann Neurol. 1986;19:105–11. 2421636 10.1002/ana.410190202

[27] Abramov AY, Scorziello A, Duchen MR. Three distinct mechanisms generate oxygen free radicals in neurons and contribute to cell death during anoxia and reoxygenation. Journal Neurosci. 2007;27:1129–38. 10.1523/JNEUROSCI.4468-06.2007 PMC6673180 17267568

[28] Liu Y, Fechter LD. MK-801 protects against carbon monoxide-induced hearing loss. Toxicol Appl Pharm. 1995;132:196–202. 10.1006/taap.1995.1099 7785048

[29] Abramov AY, Duchen MR. Impaired mitochondrial bioenergetics determines glutamate-induced delayed calcium deregulation in neurons. Biochim Biophys Acta. 2010;1800:297–304. 19695307 10.1016/j.bbagen.2009.08.002

[30] Castilho RF, Hansson O, Ward MW, Budd SL, Nicholls DG. Mitochondrial control of acute glutamate excitotoxicity in cultured cerebellar granule cells. J Neurosci. 1998;18:10277–86. 9852565 10.1523/JNEUROSCI.18-24-10277.1998 PMC6793348

[31] Shinozaki Y, Nomura M, Iwatsuki K, Moriyama Y, Gachet C, Koizumi S. Microglia trigger astrocyte-mediated neuroprotection via purinergic gliotransmission. Sci Rep. 2014;4:4329. 24710318 10.1038/srep04329 PMC3948352

[32] Kamynina A, Esteras N, Koroev DO, Angelova PR, Volpina OM, Abramov AY. Activation of RAGE leads to the release of glutamate from astrocytes and stimulates calcium signal in neurons. J Cell Physiol. 2021;236:6496–506. 33570767 10.1002/jcp.30324 PMC8651009

[33] Maiolino M, O’Neill N, Lariccia V, Amoroso S, Sylantyev S, Angelova PR, et al. Inorganic polyphosphate regulates AMPA and NMDA receptors and protects against glutamate excitotoxicity via activation of P2Y receptors. J Neurosci. 2019;39:6038–48. 31147524 10.1523/JNEUROSCI.0314-19.2019 PMC6668208

[34] Fukumoto Y, Tanaka KF, Parajuli B, Shibata K, Yoshioka H, Kanemaru K, et al. Neuroprotective effects of microglial P2Y(1) receptors against ischemic neuronal injury. J Cereb Blood Flow Metab. 2019;39:2144–56. 30334687 10.1177/0271678X18805317 PMC6827120

[35] Hettiarachchi NT, Boyle JP, Bauer CC, Dallas ML, Pearson HA, Hara S, et al. Peroxynitrite mediates disruption of Ca^2+^ homeostasis by carbon monoxide via Ca^2+^ ATPase degradation. Antioxidants redox Signal. 2012;17:744–55. 10.1089/ars.2011.4398 22360385

[36] Gunter TE, Pfeiffer DR. Mechanisms by which mitochondria transport calcium. Am J Physiol. 1990;258:C755–86. 2185657 10.1152/ajpcell.1990.258.5.C755

[37] Bernardi P, Carraro M, Lippe G. The mitochondrial permeability transition: recent progress and open questions. FEBS J. 2022;289:7051–74. 34710270 10.1111/febs.16254 PMC9787756

[38] Bonora M, Giorgi C, Pinton P. Molecular mechanisms and consequences of mitochondrial permeability transition. Nat Rev Mol Cell Biol. 2022;23:266–85. 34880425 10.1038/s41580-021-00433-y

